# Unveiling the Dual Nature of Heavy Metals: Stressors and Promoters of Phenolic Compound Biosynthesis in *Basilicum polystachyon* (L.) Moench In Vitro

**DOI:** 10.3390/plants13010098

**Published:** 2023-12-28

**Authors:** Sumanta Das, Kaniz Wahida Sultana, Moupriya Mondal, Indrani Chandra, Ashwell R. Ndhlala

**Affiliations:** 1 Department of Biotechnology, The University of Burdwan, Burdwan 713104, West Bengal, India; kwsultana@gmail.com (K.W.S.); moupriyamondal652@gmail.com (M.M.); 2 Department of Plant Production, Soil Science and Agricultural Engineering, Green Biotechnologies Research Centre of Excellence, University of Limpopo, Private Bag X1106, Sovenga 0727, South Africa

**Keywords:** abiotic stress, lamiaceae family, lead, medicinal plant, mercury, phenolic acids, propagation

## Abstract

The global industrial revolution has led to a substantial rise in heavy metal levels in the environment, posing a serious threat to nature. Plants synthesize phenolic compounds under stressful conditions, which serve as protective agents against oxidative stress. *Basilicum polystachyon* (L.) Moench is an herbaceous plant of the Lamiaceae family. Some species within this family are recognized for their capacity to remediate sites contaminated with heavy metals. In this study, the effects of mercury (II) chloride and lead (II) nitrate on the in vitro propagation of *B. polystachyon* were investigated. Shoot tips from in vitro plantlets were cultured in Murashige and Skoog’s (MS) media with heavy metals ranging from 1 to 200 µM to induce abiotic stress and enhance the accumulation of phenolic compounds. After three weeks, MS medium with 1 µM of lead (II) supported the highest shoot multiplication, and the maximum number of roots per explant was found in 100 µM of lead (II), whereas a higher concentration of heavy metals inhibited shoot multiplication and root development. The plantlets were hardened in a greenhouse with a 96% field survival rate. Flame atomic absorption spectroscopy (FAAS) was used to detect heavy metal contents in plant biomass. At both 200 µM and 50 µM concentrations, the greatest accumulation of mercury (II) was observed in the roots (16.94 ± 0.44 µg/g) and shoots (17.71 ± 0.66 µg/g), respectively. Similarly, lead (II) showed the highest accumulation in roots (17.10 ± 0.54 µg/g) and shoots (7.78 ± 0.26 µg/g) at 200 µM and 50 µM exposures, respectively. Reverse-phase high-performance liquid chromatography (RP-HPLC) identified and quantified various phenolic compounds in *B. polystachyon* leaves, including gallic acid, caffeic acid, vanillic acid, *p*-coumaric acid, ellagic acid, rosmarinic acid, and *trans*-cinnamic acid. These compounds were found in different forms, such as free, esterified, and glycosylated. Mercury (II)-exposed plants exhibited elevated levels of vanillic acid (1959.1 ± 3.66 µg/g DW), ellagic acid (213.55 ± 2.11 µg/g DW), and rosmarinic acid (187.72 ± 1.22 µg/g DW). Conversely, lead (II)-exposed plants accumulated higher levels of caffeic acid (42.53±0.61 µg/g DW) and *p*-coumaric acid (8.04 ± 0.31 µg/g DW). *Trans*-cinnamic acid was the predominant phenolic compound in control plants, with a concentration of 207.74 ± 1.45 µg/g DW. These results suggest that sublethal doses of heavy metals can act as abiotic elicitors, enhancing the production of phenolic compounds in *B. polystachyon*. The present work has the potential to open up new commercial opportunities in the pharmaceutical industry.

## 1. Introduction

Heavy metal contamination is a serious environmental threat that substantially limits crop productivity [1]. Essential metals like cobalt (Co^2+^), copper (Cu^2+^), iron (Fe^2+^), manganese (Mn^2+^), nickel (Ni^2+^) and zinc (Zn^2+^) are crucial for plant growth and development [2]. In contrast, non-essential heavy metals such as cadmium (Cd^2+^), mercury (Hg^2+^), lead (Pb^2+^), chromium (Cr^2+^), arsenic (As^2+^), etc. are extremely noxious for plants and animals [3]. High concentrations of these metals can be harmful to plants, as they interfere with plant metabolism and development [4]. The excessive accumulation of heavy metals in plant tissues can interfere with photosynthesis, respiration and nutrient uptake [5]. Both lead and mercury are the most toxic elements that can disrupt plant growth, as recognized by the United States Environmental Protection Agency (EPA) and the US Agency for Toxic Substances and Disease Registry (ATSDR) [6,7]. Mercury exists in various forms, including elemental, inorganic, and organic compounds, all of which are toxic. Lead, on the other hand, forms particularly harmful organometallic compounds when combined with carbon [8]. They can cause oxidative stress in cells, leading to increased production of reactive oxygen species (ROS), lipid peroxidation, and signaling compounds that can interrupt the defense system of plants [5,9,10].

Muszynska, et al. [11] and Demarco, et al. [12] found that some plants can survive in stressful conditions, even when high concentrations of heavy metals can cause alterations in photosynthesis by reducing the chlorophyll content in leaves. Elevated levels of heavy metals in the growth medium can impact mineral uptake, leading to imbalances between essential and trace elements, as demonstrated by Gatti [13] and Okem, et al. [14]. Interestingly, some studies have reported that low concentrations of heavy metals can promote plant growth [15,16,17,18,19]. However, prolonged exposure to heavy metals can generate free radicals like superoxide anion (O^2−^), hydroxyl radicals (.OH), and non-free radicals such as hydrogen peroxide (H_2_O_2_), organic peroxide (ROOH), and singlet oxygen (^1^O_2_) [20]. Plants respond to heavy metal stress by upregulating genes that encode proteins involved in the production of secondary metabolites [21]. The production of secondary metabolites in plants can be either stimulated or inhibited by exposure to heavy metal contamination [22]. Heavy metal exposure reduces the production of secondary metabolites in plants as per previous studies [23,24,25]. There is some evidence that plants can produce phenolic compounds in the presence of metals by increasing their metabolic activity [26,27,28,29,30,31].

The Industrial Revolution transformed global perspectives on addressing environmental waste management, as traditional remediation technologies were often costly and could have adverse effects on the environment. In contrast, plant-based remediation is a more sustainable and efficient approach that requires low energy and minimal expenses and can effectively remove metal pollutants from the environment or transform them into non-toxic forms. Non-edible aromatic plants are often a more appropriate choice for remediation efforts due to their inherent aromatic properties, minimizing the risk of contaminating the food chain with harmful substances [32]. Importantly, these plants are neither consumed by humans nor animals, effectively halting the transmission of heavy metals from soil to the food chain, and subsequently, to the human body [33]. Plant tissue culture is a powerful tool for selecting metal-tolerant plants, which can be used for phytoremediation [34,35,36,37,38,39]. A diverse range of plants, including shrubs, ornamental perennials, and annuals, can take up and degrade pollutants. Among the species of the Lamiaceae family, *Ocimum basilicum* L. holds the potential for phytoremediation [40,41]. Many other plant species can also clean up heavy metals from soil. These include *Bidens pilosa* L., *Tagetes minuta* L. [42], *Salix alba* L. [43], *Helianthus annuus* L. [44,45] for lead and *Brassica juncea* L. [41], *Caladium bicolor* L., *Cyperus kyllingia* L., *Digitaria radicosa* (Presl) Miq, *Lindernia crustacea* L., *Paspalum conjugatum* L. and *Zingiber purpureum* (Roxb.) [46] for mercury.

Furthermore, other plant species recognized for their ability to accumulate heavy metals comprise *Basilicum polystachyon* (L.) Moench, the plant selected for this study, which is a fast-growing Lamiaceae species. This aromatic herb is found in Asia, Africa, and India [47]. The present study investigated the potential for in vitro propagation of *B. polystachyon* under heavy metal stress, as well as the accumulation of heavy metals in plant biomass and the effect of heavy metal stress on the enhancement of phenolic compounds.

## 2. Materials and Methods

### 2.1. Chemicals and Solvents

Murashige and Skoog’s (MS) basal medium, agar-agar, and diluent solution for DNA were obtained from Hi-media, India. Bavestien^®^ (Carbendazimpowder) was procured from ASF India Limited, New Delhi, India. Tween-20 (Polysorbate 20), mercury (II) chloride, lead (II) nitrate, methanol, n-hexane, acetonitrile, acetone, ethyl acetate, diethyl ether, nitric acid (HNO_3_), perchloric acid (HClO_4_), sucrose, sodium hydroxide (NaOH), ethylenediaminetetraacetic acid (EDTA) and phytagel were obtained from Merck, Merck-Sigma Aldrich, St. Louis, MO, USA. All standard phenolic compounds viz., gallic acid, caffeic acid, *p*-coumaric acid, ellagic acid, rosmarinic acid, *trans*-cinnamic acid and vanillic acid were procured from Sigma Aldrich, Merck-Sigma Aldrich, St. Louis, MO, USA. All the solvents used in these experiments were HPLC grade.

### 2.2. Source of Plant Material and Sterilization Grade

The *B. polystachyon* sample was obtained from 2-month-old plants grown ex vitro in the field at the Department of Biotechnology, The University of Burdwan, Burdwan 713104, West Bengal, India (23°15′25.2″ N, 87°51′01.7″ E). The plant specimen was identified and verified by K. Karthigeyan, Scientist—‘F’, at the Botanical Survey of India in Kolkata, and a voucher specimen (BU/SD-01) was deposited at the Department of Biotechnology at The University of Burdwan. The use of this plant in the present study complies with institutional, national, and international guidelines and legislation. All experiments were performed in accordance with relevant guidelines and regulations. Shoot tips were excised from the plant and used as the explant source. Explants were washed in Milli-Q water (Millipore system, Merck, Rahway, NJ, USA) for 5 min, then disinfected in 70% ethanol *v*/*v* for 10 s followed by washing with 0.01% *v*/*v* Tween-20 for 4 min [48,49]. Surface sterilization was performed using 0.1% *w*/*v* mercuric chloride for 45 s in a Biosafety Cabinet A2 (Biobase Inc., Jinan, China) under aseptic conditions. The explant was thoroughly rinsed three times with Milli-Q water after sterilization [34,50].

### 2.3. Media Preparation and Culture Condition

A shoot tip measuring 6–7 mm in length was placed in a culture vessel (25 × 150 mm) containing 20 mL of Murashige and Skoog’s (MS) basal media, as developed by Murashige and Skoog [51]. The media was enriched with various concentrations (0, 1, 25, 50, 100, and 200 µM) of heavy metals, specifically mercury (II) chloride and lead (II) nitrate. Additionally, the media contained 30 g/L sucrose and 0.15% *w*/*v* phytagel as supporting substances. A control culture was also established using MS medium without the inclusion of heavy metals. To ensure the best environment for plant regeneration, the plant growth chamber was maintained at a constant temperature of 25 ± 2 °C, 55% humidity, and subjected to a 16 h photoperiod with 2000 lux of light intensity [34].

### 2.4. Shoot Multiplication, Rooting and Acclimatization

The multiplication of regenerated in vitro shoots was achieved by transferring mother explants and subculturing in vitro raised plantlets on fresh culture media at a regular interval of three weeks. Each experiment was repeated three times and the data were recorded after three weeks using 20 replicates. Experimental data were recorded considering parameters such as the number of shoots per explant, shoot length, number of roots per shoot, and root length. In vitro plantlets (three weeks old) were removed from culture tubes, thoroughly washed with Milli-Q water to remove agar, and then transferred to plastic pots (100 × 80 mm) containing a sterilized mixture of sand and soil (1:1 *w*/*w*). A 0.1% *w*/*v* solution of Bavestien^®^ was applied to the surface of the plastic pots to inhibit fungal growth. The pots were covered with transparent poly bags (300 × 220 mm) and placed in a plant growth chamber (Thermo Fisher Scientific, Waltham, MA, USA) at a temperature of 25 ± 2 °C with a 16 h photoperiod. After one week, the plastic covers were removed, and the plantlets were maintained under the same conditions for two weeks. The partially acclimatized plantlets were then moved to a net house environment. After two weeks, acclimatized plants were transferred to their natural habitat [34].

### 2.5. In Vitro Selection and Analysis of Heavy Metal Contents

To evaluate heavy metal accumulation in *B. polystachyon* under different concentrations, in vitro regenerated plantlets (three weeks old) were collected, washed, and subsequently dried at room temperature. The plantlets were then divided into two parts i.e., shoot and root. A 0.1 g of dried tissue sample was transferred to a Teflon-lined vessel and digested with HNO_3_-HClO_4_ (3:1 *v*/*v*) in a microwave (LG, Delhi, India) digester. After dilution with distilled water up to 25 mL, the samples were filtered and analyzed by flame atomic absorption spectroscopy (PerkinElmer Inc., Waltham, MA, USA). The linearity and range of different concentrations of reference heavy metals were evaluated precisely. The determination of metals used a specific hollow cathode lamp (HCL) and air-acetylene flame, with a slit width set to 0.7 nm and detection wavelengths of 283.3 nm and 253.7 nm for lead (II) and mercury (II), respectively [52]. The heavy metal contents in plant parts were expressed as µg/g DW.

#### Determination of Tolerance Index (*TI*) and Translocation Factor (*TF*)

The *TI* can be calculated according to the following equation [53,54]:

$$TI\;(\%)=\frac{Dry\;weight\;of\;treated\;plant}{Dry\;weight\;of\;control\;plant}\times 100$$

The *TF* was determined according to the formula followed by Yoon, et al. [55]:

$$TF=\frac{Heavy\;metal\;content\;in\;shoot}{Heavy\;metal\;content\;in\;root}$$

### 2.6. Extraction, Identification, Quantification and Assessment of Phenolic Compounds

Heavy-metal-induced and control plant cultures (50 µM) were collected. Leaves were cut, air-dried for 72 h, and ground into fine powder. Then, 0.5 g of each sample powder was dissolved in 10 mL of an extraction buffer (methanol: water: acetone in a 5:3:2 ratio) and incubated for 48 h in a shaker incubator (Spac-N-Service, Kolkata, India) at room temperature. The samples were subjected to ultrasonic extraction (PIEZO-U-SONIC Ultrasonic Processor, Kolkata, India) at two distinct time intervals, i.e., 5 min and 10 min. The frequency of the ultrasound was 40 kHz. The extracted solutions were centrifuged (CPR-30 Plus, Remi Lab World, Mumbai, India) at 5600× *g* for 5 min, and the supernatant was collected and filtered through a PTFE membrane filter (Hi-media, Thane, India). The resulting supernatant was used to separate the different forms of phenolic compounds, i.e., free, esterified and glycosylated using the method described by Das, et al. [56] and Arruda, et al. [57].

#### 2.6.1. Extraction of Free Form Phenolic Compounds

The previously obtained supernatant was subjected to rotary vacuum evaporation (RE 100 Pro, Biobase Inc., Jinan, China) at 40 °C to remove organic solvents. The resulting aqueous phase was acidified to pH 2 and transferred to a separating funnel. The clear supernatant was extracted three times with an equal volume of n-hexane (1:1, *v*/*v*) to eliminate interfering lipid molecules. The organic and aqueous phases were separated, and the aqueous phase was collected. An equal volume of diethyl ether and ethyl acetate (1:1, *v*/*v*) was added to the aqueous phase, and the mixture was transferred to a separating funnel. The organic phase was collected, dehydrated, and filtered through anhydrous sodium sulfate using Whatman No. 1 filter paper. The solvent was then removed under vacuum rotary evaporation at 35 °C. The resulting dry residue of free form phenolics was dissolved in 1 mL of methanol for further use.

#### 2.6.2. Extraction of Esterified Form Phenolic Compounds

The remaining aqueous phase, obtained from the extraction of the esterified phenolics, was hydrolyzed with a mixture of 4 M NaOH, 10 mM EDTA, and 1% ascorbic acid (using a solvent to aqueous phase ratio of 2:1, *v*/*v*). The solution was incubated for 3 h at room temperature in a water bath shaker (120 rpm) to release the esterified phenolics. The pH of the solution was adjusted to 2 and transferred to a separating funnel, where it was mixed with an equal volume of diethyl ether and ethyl acetate (1:1, *v*/*v*). The organic phase was collected, dehydrated, and filtered through anhydrous sodium sulfate using Whatman No. 1 filter paper. The resulting solution was evaporated under a vacuum using a rotary evaporator at 35 °C. The dry residue of esterified form phenolics was dissolved in 1 mL of methanol for further use.

#### 2.6.3. Extraction of Glycosylated Form Phenolic Compounds

The aqueous solution left over from the extraction of esterified phenolic compounds was subjected to hydrolysis with twice the volume of 6 M HCl and incubated for 30 min in a shaker incubator (120 rpm) to release glycosylated phenolic compounds. The pH of the resulting solution was adjusted to 2 and then mixed with an equal volume of diethyl ether and ethyl acetate (1:1, *v*/*v*) in a separating funnel. The organic phase was collected, dehydrated, and filtered using Whatman No. 1 filter paper with anhydrous sodium sulfate. The resulting solution was evaporated under a vacuum rotary evaporator at 35 °C. The dry residue of glycosylated form phenolics was dissolved in 1 mL of methanol for future use.

### 2.7. Instrumentation

An RP-HPLC system (Chromaster, Hitachi Corporation, Tokyo, Japan) equipped with a UV detector and a quaternary gradient pump was utilized for the analysis of phenolic compounds. The C18 reversed-phase column (5C18-MS-II, 4.6 ID 250 mm, cosmosil- Nacalai Tesque INC., Kyoto, Japan) was maintained at a temperature of 25 °C. RP-HPLC is a widely used technique for analyzing phenolic compounds, and most of these compounds can be detected in the UV range [58]. The elution gradient for the sample was achieved by using two solvents: solvent A (2% glacial acetic acid in water) and solvent B (acetonitrile: water, 70:30) (Supplementary Table S1). A 20 µL sample was injected, and the flow rate was set to 1 mL/min. The detection wavelength was 280 nm [59].

#### Identification and Quantification of Phenolic Compounds Using RP-HPLC

Phenolic compounds were identified by comparing the retention times of extracted compounds with those of standard phenolic acids, including gallic acid, caffeic acid, *p*-coumaric acid, rosmarinic acid, *trans*-cinnamic acid, vanillic acid, and ellagic acid. Standard solutions of each acid were prepared by dissolving 1 mg in 1 mL of methanol and were used to quantify the phenolic compounds present in the leaves of *B. polystachyon*. The quantitative determination of phenolic compounds was carried out following the proposed method [50,60].

$$Sample\;concentration(µg/gDW)=\frac{Sample\;area}{Standard\;area}\times \frac{Standard\;weight}{Standard\;dilution}\times \frac{Sample\;dilution}{Sample\;weight}$$

### 2.8. Data Collection and Statistical Analysis

The data were analyzed using one-way analysis of variance (ANOVA) and the results were presented as mean ± standard error. The significance of differences among means was determined by Duncan’s multiple range test (DMRT) [61,62] at a significance level of *p* ≤ 0.05 using SPSS 26.0 version software (SPSS Inc., Armonk, NY, USA). RP-HPLC data and statistical graphs were generated using Origin 2022 and GraphPad Prism 9.5, respectively.

## 3. Results and Discussion

### 3.1. Effect of Heavy Metal on In Vitro Propagation and Tolerance Index

**Figure 1:**
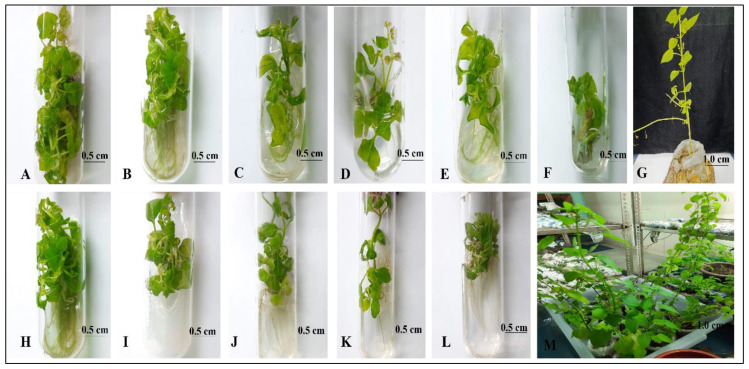
Effect of heavy metal stress on in vitro propagation of *B. polystachyon* after three weeks of culture incubation. (**A**) Control, (**B**) MS medium containing 1 µM Hg (II), (**C**) 25 µM Hg (II), (**D**) 50 µM Hg (II), (**E**) 100 µM Hg (II), (**F**) 200 µM Hg (II), (**G**) acclimatization of plantlets, (**H**) 1 µM Pb (II), (**I**) 25 µM Pb (II), (**J**) 50 µM Pb (II), (**K**) 100 µM Pb (II), (**L**) 200 µM Pb (II), (**M**) hardened plantlets.

Shoot tips from field-grown plants of *B. polystachyon* were cultured in MS media that contained various concentrations of mercury (II) and lead (II) for plant regeneration (Table 1 and Table 2). Multiple shoots with roots were induced after 3 weeks of culture incubation in MS media that was supplemented with various concentrations of mercury (II) (Figure 1B–F). Plants showed tolerance to all concentrations of heavy metals (1, 25, 50, 100, and 200 µM), as evidenced by the lack of chlorosis and necrosis. Heavy metal concentration significantly impacted plant growth in *B. polystachyon*, with lower concentrations promoting better shoot and root development (Table 1 and Figure 1). Among the concentrations tested, 1 µM mercury (II) induced the highest number and length of shoots in *B. polystachyon* (Figure 1A,B). Increasing the concentration reduced overall growth, resulting in a gradual decline in both shoot and root lengths. Surprisingly, at 50 µM, root number was maximized while maintaining moderate shoot growth, as depicted in Figure 1D. However, the shoot length was greatly reduced in 100 µM concentration of mercury (II), and root formation was generally poor (Figure 1E). In addition, stout and hairy adventitious roots were observed in MS media supplemented with 200 µM of mercury (II) (Figure 1F). This highlights the complex interplay between heavy metal concentration and plant growth, suggesting the existence of a critical balance for optimal development. The present results are consistent with [63] reports that higher concentrations of mercury (II) inhibit the growth of *Pisum sativum* L. Passow and Rothstein [64] and Shieh and Barber [65] also reported that mercury (II) affected cell membranes, leading to a breakdown in the transport mechanism of plants. Higher concentrations of heavy metal stress resulted in increased leaf wilting, significantly reduced shoot multiplication, and shorter shoots and roots compared to control plants. This could potentially be due to a reduction in cell division and differentiation [66]. Several studies on basil plants suggest that the presence of heavy metals in the growth medium has negative effects on various physiological processes [67,68].

As depicted in Figure 1H–L, the results suggest that higher concentrations of lead (II) had a more significant impact on in vitro regeneration. Lead (II) at 1 μM in MS media maximized both shoot multiplication and root induction (Figure 1H). Although 50 μM proved optimal for both, exceeding this level generally inhibited root development, with a notable exception at 100 μM, where root number unexpectedly increased (Figure 1K). Intriguingly, at 200 μM, the mean shoot length plummeted to 1.00 ± 0.40 cm (Figure 1L). Significantly, shoot multiplication was completely suppressed at 200 μM of both mercury (II) and lead (II), whereas robust adventitious root formation persisted. Intriguingly, the presence of these metals also induced *B. polystachyon* to flower, as shown in Figure 1D. This finding aligns with the previously reported observation that heavy metals can promote flowering, suggesting a more complex interplay between these elements and plant development [69]. Similar to *Cyamopsis tetragonoloba* L. and *Sesamum indicum* L. [70], which exhibited tolerance to lead (II), *B. polystachyon* also demonstrated the ability to tolerate low levels of heavy metals, according to this study. Heavy metal stress, particularly at high levels, significantly reduced plant growth and development, likely due to interference with nutrient uptake pathways. The present study demonstrates that in vitro-regenerated *B. polystachyon* plantlets exhibit a noteworthy degree of tolerance to heavy metals.

Acclimatization of in vitro-grown plantlets was essential for their successful transplantation to in vivo climatic conditions. By the end of the experiment, the result established that *B. polystachyon* plantlets exposed to heavy metals were successfully propagated. Subsequently, the plantlets were acclimatized to the greenhouse environment, and the hardened plants were transplanted to the field with a 96% survival rate (Figure 1G–M). Based on the findings, it can be demonstrated that the in vitro propagation technique offers a promising approach for selecting heavy-metal-tolerant plants.

As shown in Table 1 and Table 2, the results demonstrated that as the plant was exposed to different concentrations of heavy metals, the TI (tolerance index) values markedly decreased at higher concentrations. Specifically, the plant had a 70.86% TI at 100 µM of lead (II) and a 42.18% TI at 200 µM of lead (II). Similarly, the plant had a 78.75% TI at 50 µM of mercury (II) and a 40% TI at 200 µM of mercury (II). Interestingly, low concentrations of mercury (II) and lead (II) had a partially negative effect on shoot and root growth, but *B. polystachyon* was capable of accumulating high levels of heavy metals and had a survival rate of 96%. Cano-Ruiz, et al. [71] found similar results for cadmium, nickel, lead, and copper. This study supports the work of Youssef [72] on *Ocimum basilicum* L., which confirmed the effects of heavy metals on the plant. Other studies have shown that plants from the Lamiaceae family, such as *Mentha crispa* L., *Mentha piperita* L, *Ocimum basilicum* L. and *Ocimum sanctum* L. can exhibit resistance against the harmful effects of heavy metal toxicity [73,74,75]. The results of the study suggest that plantlets regenerated in vitro could be used to accumulate heavy metals in contaminated sites.

**Figure 2:**
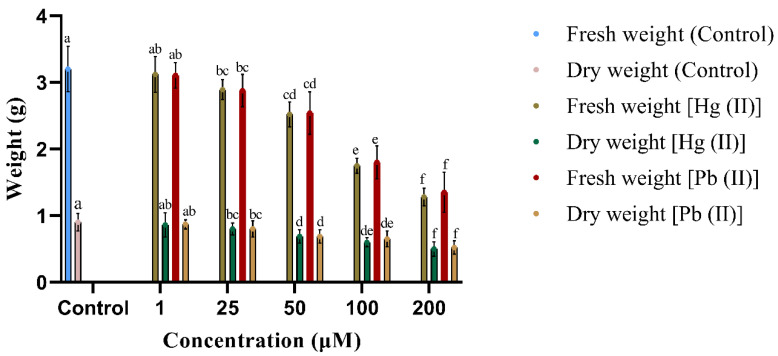
Effect of heavy metals on the fresh and dry weights of in vitro-raised plantlets. Values represent mean ± SE of 10 replicates per experiment, with each experiment repeated three times. Mean followed by the same letter is not significantly different (*p* ≤ 0.05) using Duncan’s multiple range test.

The effect of varying concentrations of heavy metals on *B. polystachyon* was assessed by measuring the fresh and dry weights of plantlets regenerated using the in vitro method. As the concentrations of mercury (II) and lead (II) increased in the MS media, the fresh and dry weights of the plantlets gradually decreased, as shown in Figure 2. The highest fresh weight (3.12 ± 0.27 g) and dry weight (0.86 ± 0.18 g) of *B. polystachyon* were noted in the MS media supplemented with 1 µM of mercury (II) compared to lead (II), but the difference was not statistically significant. The fresh and dry weights of plantlets decreased as the concentration of heavy metals increased in MS media. The plant biomass declined significantly at 100 µM and 200 µM, after reaching 50 µM. Figure 2 illustrates that, despite the control plants exhibiting marginally greater mean fresh weight (3.2 ± 0.34 g) and dry weight (0.9 ± 0.13 g), there was no significant difference in biomass between the control and the 1 µM heavy metal treatments. These findings suggest that low concentrations of heavy metals did not significantly affect the plant biomass.

Moreover, a reduction in the biomass of in vitro regenerated plantlets was noted in the regenerating media with higher concentrations of heavy metals. The growth of the plant was significantly affected by the addition of 200 µM of mercury (II) and lead (II) in the medium, as evidenced by the significant reduction in shoot and root length. The toxic nature of heavy metals interferes with cellular levels, disrupting the plant metabolic pathways and ultimately reducing growth and development. Previous research demonstrated that high concentrations of heavy metals in the growth medium can disrupt the ability of plants to take up water and nutrients, leading to decreased biomass in *Ocimum basilicum* L. and *Mentha piperita* L [33,76,77].

### 3.2. Potential for Heavy Metal Accumulation

**Figure 3:**
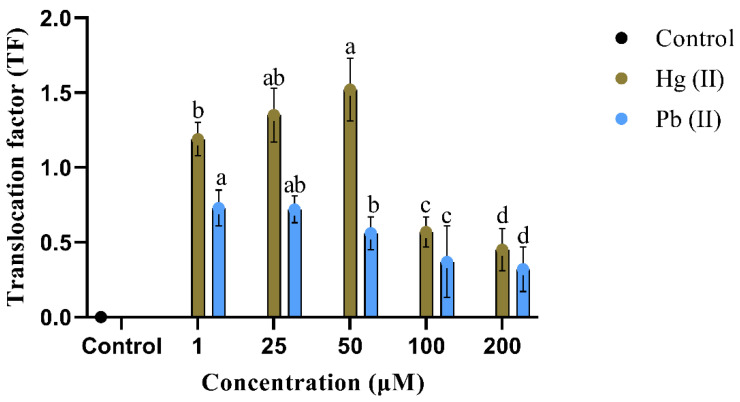
TF of Hg (II) and Pb (II) in *B. polystachyon*. Values represent mean ± SE of 3 replicates per experiment, with each experiment repeated three times. Mean followed by the same letter is not significantly different (*p* ≤ 0.05) using Duncan’s multiple range test.

In Table 3, it was demonstrated that the plant exhibited the ability to accumulate a particular level of heavy metals in both its root and shoot. Notably, the highest accumulation of mercury (II) was observed at concentrations of 200 μM and 50 μM, with the root and shoot values of 16.94 ± 0.44 μg/g and 17.68 ± 0.66 μg/g, respectively. There was no significant difference observed in the accumulation of heavy metal in the shoot between concentrations of 1 µM and 100 µM. Figure 3 shows the TF values for heavy metals, where values greater than one indicate efficient metal translocation from root to shoot.

The present study found that the roots of *B. polystachyon* accumulated the highest level of mercury (II) at a concentration of 200 μM, with a translocation factor of 0.45. However, at a concentration of 50 μM, the TF value was 1.52, suggesting that the mercury (II) was able to move from the root into the shoot. Lone, et al. [78] reported that the accumulation of high levels of metals in aerial parts of the plant led to decreased plant height, which is consistent with a previous study on *Lindernia crustacea* L., where the maximum mercury accumulation occurred in the shoots [46]. Additionally, it was stated that the TF can be influenced by various factors, such as plant species, root uptake efficiency, water absorption, element type, and soil nutrient availability.

The accumulation capacity of *B. polystachyon* showed that the plant could survive heavy metal stress up to 200 μM. The root accumulated significantly higher lead (II) than the shoot, with concentrations of 17.10 ± 0.54 μg/g and 7.78 ± 0.26 μg/g, respectively, at 200 μM and 50 μM. The results also revealed that increasing the concentration of lead (II) led to a decrease in TF values, suggesting that the plant was less able to transport lead (II) from the roots into the shoots at higher concentrations. The lowest TF value (0.32) was found in lead (II) enriched plants, indicating that the majority of the lead (II) remained in the roots and was not translocated to the shoots. Previous studies have shown that lead (II) accumulates mostly in the roots, gradually moving to the shoots [79,80]. The study found that the plant had the potential to accumulate and translocate lead (II) and mercury (II) in root and shoot. Purohit, et al. [81] and Yan, et al. [82] demonstrated that the transporter of P1B-type ATPases is involved in the transport of heavy metals from root to shoot. The results of this study suggest that *B. polystachyon* is a potential plant for phytoremediation, even though it accumulates high levels of lead (II) in its roots.

Several studies have shown that lead (II) primarily accumulates in the roots of plants due to its binding to ion-exchangeable sites on the cell wall, which prevents it from moving into the cells [83,84]. The present results are consistent with previous studies that have shown that lead (II) is poorly translocated from root to shoot, likely due to the presence of a physical barrier in the root zone. Plants have developed effective mechanisms to deal with high levels of heavy metal exposure, which are dependent on their biochemical processes. The present findings align with earlier studies conducted by Dinu, et al. [85] and Youssef [72], indicating that species of the Lamiaceae family, such as *Ocimum* sp., are capable of accumulating heavy metals. Additionally, these plants exhibit a higher concentration of secondary metabolites, which offer protection against oxidative damage and prevent cell oxidation. The results suggest that the plant holds promising potential for utilization in heavy metal remediation purposes.

### 3.3. Effect of Heavy Metal on the Enhancement of Production of Phenolic Compounds

Table 4 shows the contents of phenolic compounds in the leaves of plants exposed to heavy metals and the control. Supplementary Figure S1 displays the chromatograms depicting the standard phenolic acids. The results showed that there were significant variations in the contents of phenolic compounds, depending on their form. These forms included free, esterified, and glycosylated phenolics. Remarkably, the results suggest that plants grown in vitro in the presence of heavy metals may increase their production of phenolic compounds. Quantitative analysis of free, esterified, and glycosylated phenolic compounds revealed a substantial upregulation in lead (II)-exposed plants. Table 4 illustrates a significant increase in the content of free phenolics, which constitute the primary form of phenolic compounds in plants exposed to lead (II). Rosmarinic acid (30.82 ± 0.31 µg/g DW), caffeic acid (25.57 ± 0.54 µg/g DW), gallic acid (16.23 ± 0.43 µg/g DW), and ellagic acid (10.71 ± 0.22 µg/g DW) were identified as the most abundant free phenolics, whereas caffeic acid (42.53 ± 0.61 µg/g DW), gallic acid (15.95 ± 0.38 µg/g DW), and rosmarinic acid (31.13 ± 0.47 µg/g DW) dominated the esterified and glycosylated fractions, respectively (Supplementary Figure S2A–C). In addition to rosmarinic acid, *p*-coumaric acid was also found in free and glycosylated forms. Among phenolic fraction, caffeic acid (42.53 ± 0.61 µg/g DW) emerged as the most predominant phenolic compound, followed by rosmarinic acid (31.13 ± 0.47 µg/g DW), gallic acid (16.23 ± 0.43 µg/g DW), ellagic acid (12.86 ± 0.28 µg/g DW), *p*-coumaric acid (8.04 ± 0.31 µg/g DW), *trans*-cinnamic acid (7.57 ± 0.20 µg/g DW), and vanillic acid (0.54 ± 0.7 µg/g DW).

In plants exposed to mercury (II), vanillic acid was the most abundant phenolic compound, followed by ellagic acid, rosmarinic acid, *trans*-cinnamic acid, gallic acid, caffeic acid, and *p*-coumaric acid (Supplementary Figure S3). The esterified phenolics were the most abundant, followed by the glycosylated form and then the free form (Table 4). Within the esterified phenolics, vanillic acid exhibited the highest concentration (1959.1 ± 3.66 µg/g DW), followed by rosmarinic acid (187.72 ± 1.22 µg/g DW). Lower concentrations were observed for *trans*-cinnamic acid (9.30 ± 0.66 µg/g DW) and ellagic acid (14.94 ± 0.35 µg/g DW) (Supplementary Figure S3B). In the free phenolic compounds, ellagic acid emerged as the most abundant with a concentration of 213.55 ± 0.15 µg/g DW, followed by vanillic acid (77.74 ± 1.08 µg/g DW). *Trans*-cinnamic acid and gallic acid were also present but at lower concentrations of 33.046 ± 0.69 µg/g DW and 5.183 ± 0.56 µg/g DW, respectively (Supplementary Figure S3A). The glycosylated form exhibited the highest phenolic compound diversity, identifying a total of six phenolic acids, including rosmarinic acid (45.09 ± 0.78 µg/g DW), *trans*-cinnamic acid (33.32 ± 0.32 µg/g DW), gallic acid (33.16 ± 0.68 µg/g DW), caffeic acid (18.51 ± 0.44 µg/g DW), ellagic acid (7.17 ± 0.22 µg/g DW), and *p*-coumaric acid (2.13 ± 0.15 µg/g DW) (Supplementary Figure S3C). In the free and glycosylated forms, rosmarinic acid emerged as the predominant phenolic compound, with the exception of the esterified form, where ellagic acid was present. Notably, *p*-coumaric acid was exclusively detected in its glycosylated form. These findings are consistent with previous research, which has shown that plants grown in media containing heavy metals accumulate higher levels of phenolic acids [86,87].

Mercury (II) was the most effective metal in stimulating the production of vanillic acid, ellagic acid and rosmarinic acid, whereas lead (II)-induced plants exhibited different types of phenolic compounds, with similar concentrations of some phenolic acids. The results suggest that the decrease in the amount of *trans*-cinnamic acid that plants accumulate may be attributed to their exposure to lead (II) and mercury (II), which is a precursor to other phenolic compounds. Therefore, its reduction may have led to a decrease in the production of these compounds, including caffeic acid and *p*-coumaric acid. The findings of this study suggest that heavy metal exposure can lead to an increase in the production of phenolic compounds in plants which could be an adaptive response that helps plants to protect themselves from the harmful effects of heavy metals.

Mercury (II) exposure significantly enhanced the synthesis of gallic acid, vanillic acid, ellagic acid, rosmarinic acid and *trans*-cinnamic acid in *B. polystachyon* plantlets. Interestingly, lead (II) exposure resulted in lower levels of these phenolic compounds compared to mercury (II) treatment, with the exception of caffeic acid and *p*-coumaric acid, even though both metals induce the biosynthesis of phenolic acid. Lead (II)-induced plants exhibited a diverse range of phenolic compounds, although some phenolic acids were found in similar concentrations. The present findings are in line with earlier research, suggesting that elicitors, known for triggering an immune response in plants, can also enhance the production of phenolic compounds [88,89]. Kisa, et al. [90] demonstrated that heavy metal stress can alter the expression of genes involved in the production of phenylpropanoids, leading to the accumulation of large amounts of phenolic acids in stressed plants. To the best of our knowledge, this is the first report to establish that heavy metal stress significantly increases the accumulation of phenolic compounds in *B. polystachyon*.

In the control plants, the glycosylated form emerged as the predominant type of phenolic compound. Notably, *trans*-cinnamic acid (207.74 ± 1.45 µg/g DW) and gallic acid (15.52 ± 0.20 µg/g DW) were identified as the major compounds within this form (Supplementary Figure S4C). Comparatively, the free form of phenolics was found in lower concentrations than the glycosylated form, as outlined in Table 4. In the free form, vanillic acid (143.57 ± 1.7 µg/g DW), *trans*-cinnamic acid (11.52 ± 0.29 µg/g DW), and gallic acid (7.46 ± 0.24 µg/g DW) were detected (Supplementary Figure S4A), whereas the esterified form contained *trans*-cinnamic acid (82.31 ± 1.13 µg/g DW) and gallic acid (7.06 ± 0.45 µg/g DW) (Supplementary Figure S4B). *Trans*-cinnamic acid remained the major phenolic compound in both the esterified and glycosylated forms, whereas vanillic acid dominated the free form. Notably, the results indicated an increase in vanillic acid content in plants exposed to heavy metals compared to the control. This phenomenon could be attributed to the upregulation of enzymes involved in the conversion of benzoic acid to vanillic acid under stressful environmental conditions [91]. The results of this study are in line with the previous report on *Zea mays* L. plants where the content of vanillic acid increased with exposure to mercury (II) and lead (II) [90]. It is worth noting that cinnamic acid and its derivatives have applications in the pharmaceutical industry [92]. Previous research has demonstrated that rosmarinic acid processes anti-inflammatory, antiviral, antibacterial, antioxidant, and antimutagenic properties [93,94,95]. Caffeic acid has been identified as an anti-inflammatory agent [96], and a study conducted on diabetic mice in 2009 found that it could potentially combat diabetes and increase blood insulin levels [97,98]. Vanillic acid is used as a flavoring agent and also exhibits beneficial biological activities, particularly in chemo-protection, anti-inflammation, and antimicrobial activities [99]. The identified phenolic compounds from this study have the possibility for beneficial applications in the pharmaceutical and cosmetic industries.

## 4. Conclusions

To the best of our knowledge, this is the first report that presents a novel and promising approach for regenerating *B. polystachyon* in vitro in the presence of mercury (II) and lead (II). It establishes the plant’s capacity to efficiently accumulate, translocate, and adapt to these heavy metal contaminants. The findings of this study suggest that *B. polystachyon* may have the potential for heavy metal remediation, emphasizing the need for further investigation. Furthermore, the study highlights the plant’s response to heavy metal stress, which triggers an increase in the accumulation of various phenolic compounds, such as gallic acid, caffeic acid, vanillic acid, *p*-coumaric acid, ellagic acid, and rosmarinic acid. Based on the findings of the present study, it can be concluded that *B. polystachyon* possesses substantial potential for the large-scale production of a diverse range of phenolic compounds, with promising applications in various industries.

## Figures and Tables

**Table 1:** Effect of Hg (II) on in vitro shoot multiplication, root induction, and plant tolerance index.

Hg (II) (µM)	No. of Shoots Per Explant Mean ± SE	Shoot Length (cm) Mean ± SE	No. of Roots Per Shoot Mean ± SE	Root Length (cm) Mean ± SE	TI (%)
Control	8.25 ± 0.21 ^a^	4.25 ± 0.27 ^a^	8.00 ± 0.75 ^d^	5.25 ± 0.18 ^a^	0.00
1	7.50 ± 0.18 ^b^	3.25 ± 0.27 ^b^	8.25 ± 0.54 ^c^	4.75 ± 0.17 ^b^	96.87
25	6.25 ± 0.27 ^c^	2.75 ± 0.45 ^c^	8.5 ± 0.64 ^b^	4.00 ± 0.24 ^c^	90.31
50	5.25 ± 0.25 ^d^	2.25 ± 0.44 ^d^	9.0 ± 0.85 ^a^	3.25 ± 0.35 ^d^	78.75
100	2.00 ± 0.16 ^e^	2.0 ± 0.85 ^e^	4.50 ± 0.34 ^e^	2.75 ± 0.40 ^e^	54.68
200	1.00 ± 0.25 ^f^	1.75 ± 0.27 ^f^	2.50 ± 0.25 ^f^	1.75 ± 0.81 ^f^	40.00

Values represent mean ± SE of 20 replicates per experiment, with each experiment repeated three times. Mean followed by the same letter is not significantly different (*p* ≤ 0.05) using Duncan’s multiple range test.

**Table 2:** Effect of Pb (II) on in vitro shoot multiplication, root induction, and plant tolerance index.

Pb (II) (µM)	No. of Shoots Per Explant Mean ± SE	Shoot Length (cm) Mean ± SE	No. of Roots Per Shoot Mean ± SE	Root Length (cm) Mean ± SE	TI (%)
Control	8.25 ± 0.21 ^a^	4.25 ± 0.27 ^a^	8.00 ± 0.75 ^e^	5.25 ± 0.18 ^a^	0.00
1	7.75 ± 0.22 ^b^	3.50 ± 0.28 ^b^	8.25 ± 0.74 ^d^	4.0 ± 0.27 ^b^	97.18
25	6.50 ± 0.27 ^c^	2.75 ± 0.24 ^c^	9.00 ± 0.34 ^c^	3.25 ± 0.22 ^c^	90.00
50	5.75 ± 0.24 ^d^	2.25 ± 0.55 ^d^	9.5 ± 0.24 ^b^	2.50 ± 0.25 ^d^	79.37
100	2.75 ± 0.36 ^e^	1.25 ± 0.65 ^e^	10.25 ± 0.66 ^a^	2.0 ± 0.20 ^e^	70.86
200	1.75 ± 0.25 ^f^	1.0 ± 0.40 ^f^	4.00 ± 0.28 ^f^	1.0 ± 0.11 ^f^	42.18

Values represent mean ± SE of 20 replicates per experiment, with each experiment repeated three times. Mean followed by the same letter is not significantly different (*p* ≤ 0.05) using Duncan’s multiple range test.

**Table 3:** Translocation of heavy metals in various plant tissues.

Concentration (μM)	Hg (II) (μg/g)		Pb (II) (μg/g)	
	Root	Shoot	Root	Shoot
Control	0 ± 0	0 ± 0	0 ± 0	0 ± 0
1	7.34 ± 0.47 ^e^	8.76 ± 0.28 ^cd^	7.2 ± 0.19 ^e^	5.27 ± 0.43 ^de^
25	9.17 ± 0.38 ^d^	12.45 ± 0.45 ^b^	9.56 ± 0.33 ^d^	6.89 ± 0.55 ^b^
50	11.56 ± 0.56 ^c^	17.68 ± 0.66 ^a^	13.66 ± 0.64 ^c^	7.78 ± 0.26 ^a^
100	14.43 ± 0.58 ^b^	8.28 ± 0.47 ^cd^	16.48 ± 0.72 ^b^	6.24 ± 0.32 ^c^
200	16.94 ± 0.44 ^a^	7.78 ± 0.26 ^d^	17.10 ± 0.54 ^a^	5.56 ± 0.14 ^de^

Values represent mean ± SE of 3 replicates per experiment, with each experiment repeated three times. Mean followed by the same letter is not significantly different (*p* ≤ 0.05) using Duncan’s multiple range test.

**Table 4:** Comparison of phenolic compound content in *B. polystachyon* leaves under heavy metal exposure and in control plants.

Phenolic Compound	Hg (II)			Pb (II)			Control		
	Free Form Phenolics (µg/g DW)	Esterified Form Phenolics (µg/g DW)	Glycosylated Form Phenolics (µg/g DW)	Free Form Phenolics (µg/g DW)	Esterified Form Phenolics (µg/g DW)	Glycosylated Form Phenolics (µg/g DW)	Free Form Phenolics (µg/g DW)	Esterified Form Phenolics (µg/g DW)	Glycosylated Form Phenolics (µg/g DW)
Gallic acid	5.18 ± 0.56 ^f^	0 ± 0	33.16 ± 0.68 ^a^	16.23 ± 0.43 ^b^	15.95 ± 0.38 ^bc^	0 ± 0	7.46 ± 0.24 ^d^	7.06 ± 0.45 ^de^	15.52 ± 0.20 ^bc^
Caffeic acid	0 ± 0	0 ± 0	18.51 ± 0.44 ^c^	25.57 ± 0.54 ^b^	42.53 ± 0.61 ^a^	5.98 ± 0.37 ^d^	0 ± 0	0 ± 0	0 ± 0
Vanillic acid	77.74 ± 1.08 ^c^	1959.1 ± 3.66 ^a^	0 ± 0	0 ± 0	0.54 ± 0.07 ^d^	0 ± 0	143.57 ± 1.7 ^b^	0 ± 0	0 ± 0
*p*-Coumaric acid	0 ± 0	0 ± 0	2.13 ± 0.15 ^b^	8.04 ± 0.31 ^a^	0 ± 0	0.94 ± 0.05 ^c^	0 ± 0	0 ± 0	0 ± 0
Ellagic acid	213.55 ± 2.11 ^a^	14.94 ± 0.35 ^b^	7.17 ± 0.22 ^e^	10.71 ± 0.22 ^d^	12.86 ± 0.28 ^c^	4.45 ± 0.17 ^f^	0 ± 0	0 ± 0	0 ± 0
Rosmarinic acid	0 ± 0	187.72 ± 1.22 ^a^	45.09 ± 0.78 ^b^	30.82 ± 0.45 ^cd^	0 ± 0	31.13 ± 0.47 ^cd^	0 ± 0	0 ± 0	0 ± 0
*Trans*-cinnamic acid	33.046 ± 0.69 ^c^	9.30 ± 0.66 ^e^	33.32 ± 0.32 ^c^	7.57 ± 0.20 ^f^	1.61 ± 0.12 ^g^	2.15 ± 0.11 ^f^	11.52 ± 0.29 ^d^	82.31 ± 1.13 ^b^	207.74 ± 1.45 ^a^

Values represent mean ± SE of 3 replicates per experiment, with each experiment repeated three times. Mean followed by the same letter is not significantly different (*p* ≤ 0.05) using Duncan’s multiple range test.

## Data Availability

All data generated or analyzed during this study are available in the published article and its Supplementary Materials files.

## Supplementary Materials

The following supporting information can be downloaded at: https://www.mdpi.com/article/10.3390/plants13010098/s1.
