# Supplementary material for: Unveiling the Dual Nature of Heavy Metals: Stressors and Promoters of Phenolic Compound Biosynthesis in Basilicum polystachyon (L.) Moench In Vitro
Source: Plants (Basel). 2023 Dec 28;13(1):98. doi: 10.3390/plants13010098 (PMC10780674; doi:10.3390/plants13010098)
Supplement: Supplementary file 1 [file plants-13-00098-s001.zip › plants-2774921-supplementary.pdf]

# Unveiling the Dual Nature of Heavy Metals: Stressors and Promoters of Phenolic Compound Biosynthesis in *Basilicum polystachyon* (L.) Moench In Vitro

**Sumanta Das [1,*], Kaniz Wahida Sultana [1], Moupriya Mondal [1], Indrani Chandra [1,*] and Ashwell R. Ndhlala [2]**

[1] Department of Biotechnology, The University of Burdwan, Burdwan 713104, West Bengal, India; kwsultana@gmail.com (K.W.S.); moupriyamondal652@gmail.com (M.M.)
[2] Department of Plant Production, Soil Science and Agricultural Engineering, Green Biotechnologies Research Centre of Excellence, University of Limpopo, Private Bag X1106, Sovenga 0727, South Africa
* Correspondence: sumantad1991@gmail.com (S.D.); ichandrabiotech@gmail.com (I.C.)

**Supplementary Table S1.** Program for gradient elution using a mobile phase consisting of (A) 2% glacial acetic acid in water and (B) acetonitrile: water (70:30).

| Time (min) | Solvent A (2% Glacial acetic Acid in Water) | Solvent B (Acetonitrile: Water) 70:30 |
|---|---|---|
| 0-3 | 95 | 5 |
| 3-10 | 80 | 20 |
| 10-20 | 60 | 40 |
| 20-30 | 20 | 80 |
| 30-50 | 95 | 5 |

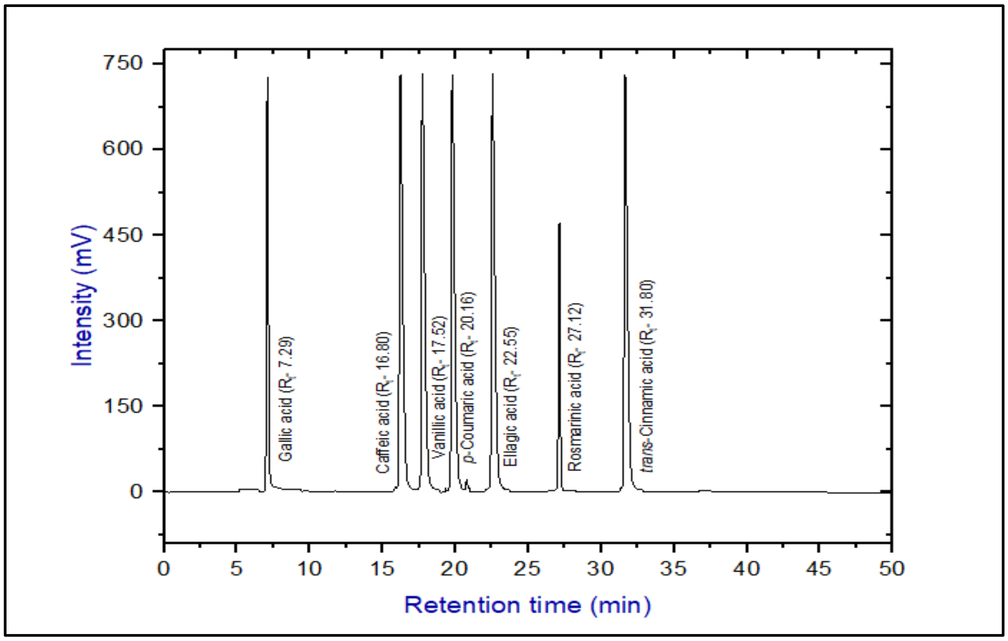


**Supplementary Figure S1.** HPLC chromatogram of standard phenolic acids: Gallic acid ($R_t$-7.29), Caffeic acid ($R_t$-16.80), Vanillic acid ($R_t$-17.52), *p*-Coumaric acid ($R_t$-20.16), Ellagic acid ($R_t$-22.55), Rosmarinic acid ($R_t$-27.12), *trans*-Cinnamic acid ($R_t$-31.80).

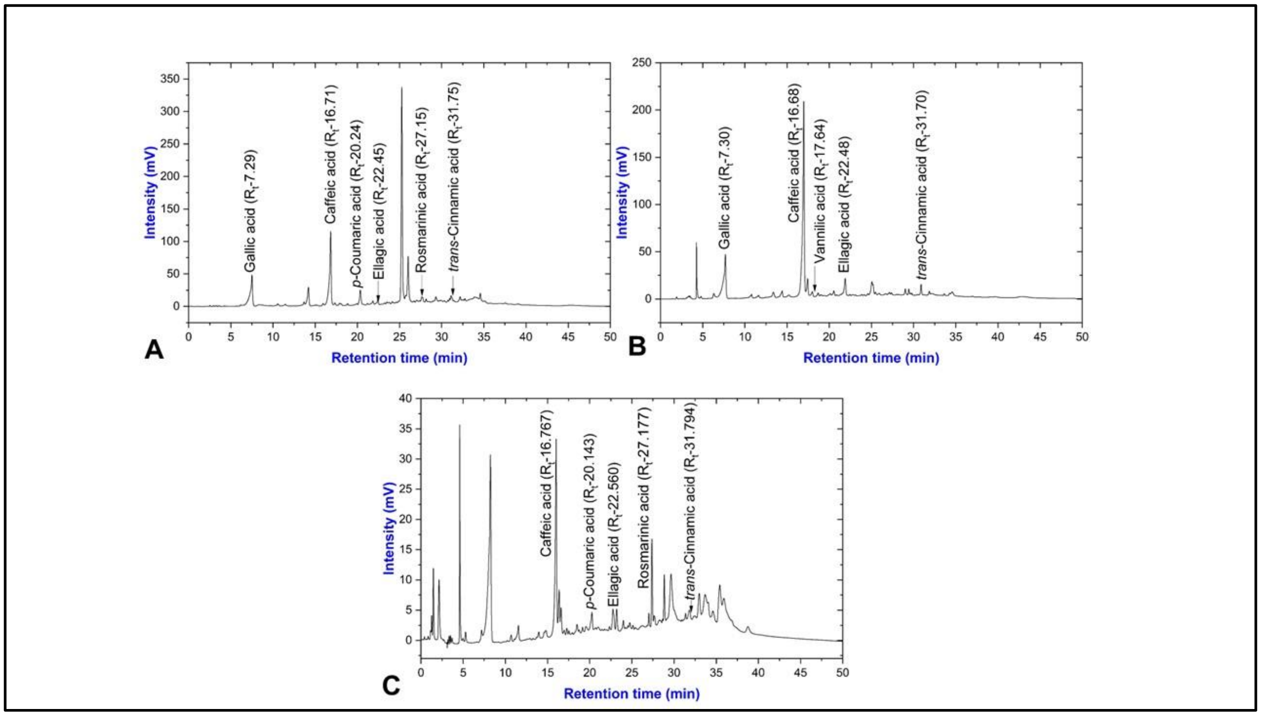


**Supplementary Figure S2.** HPLC chromatographic profile of phenolic compounds obtained in the leaves of lead (II) induced plant. (**A**) Free form phenolics, (**B**) Esterified form phenolics, and (**C**) Glycosylated form phenolics.

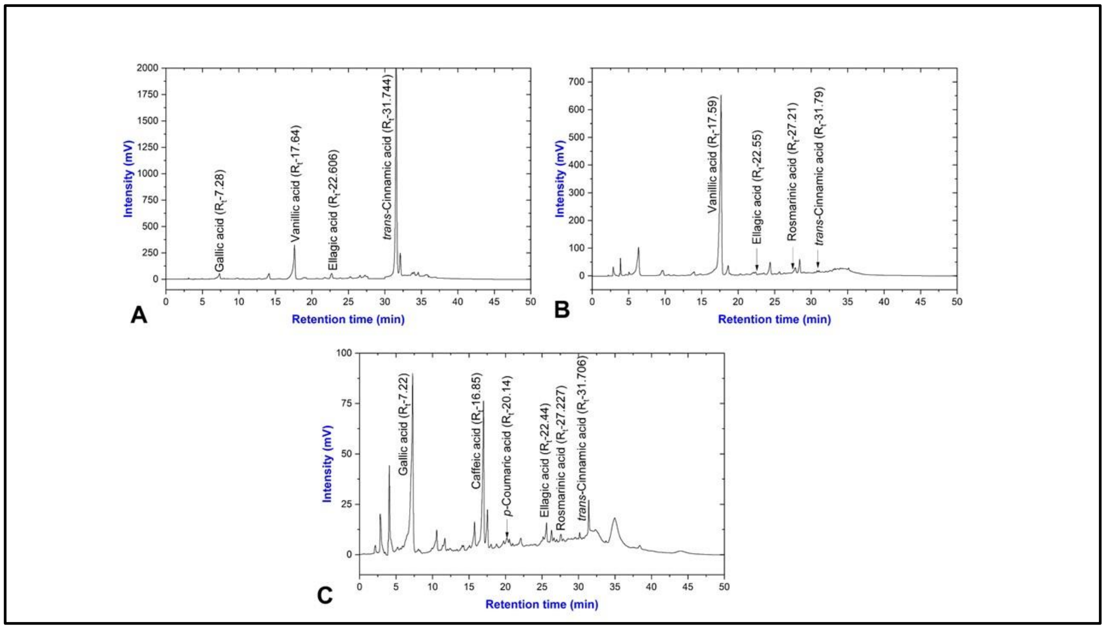


**Supplementary Figure S3.** HPLC chromatographic profile of phenolic compounds obtained in the leaves of mercury (II) induced plant. (**A**) Free form phenolics, (**B**) Esterified form phenolics, and (**C**) Glycosylated form phenolics.

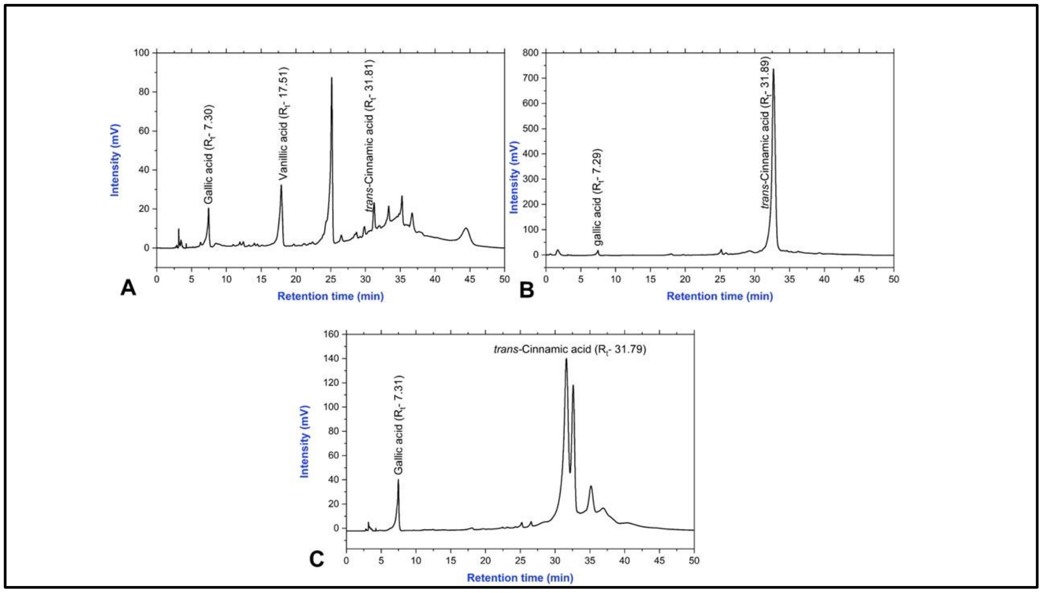


**Supplementary Figure S4.** HPLC chromatographic profile of phenolic compounds obtained in the leaves of the control plant. (**A**) Free form phenolics, (**B**) Esterified form phenolics, and (**C**) Glycosylated form phenolics**.**
